# Rocker: Open source, easy-to-use tool for AUC and enrichment calculations and ROC visualization

**DOI:** 10.1186/s13321-016-0158-y

**Published:** 2016-09-07

**Authors:** Sakari Lätti, Sanna Niinivehmas, Olli T. Pentikäinen

**Affiliations:** Computational Bioscience Laboratory, Department of Biological and Environmental Science and Nanoscience Center, University of Jyväskylä, P.O. Box 35, 40014 Jyväskylä, Finland

## Abstract

**Abstract:**

Receiver operating characteristics (ROC) curve with the calculation of area under curve (AUC) is a useful tool to evaluate the performance of biomedical and chemoinformatics data. For example, in virtual drug screening ROC curves are very often used to visualize the efficiency of the used application to separate active ligands from inactive molecules. Unfortunately, most of the available tools for ROC analysis are implemented into commercially available software packages, or are plugins in statistical software, which are not always the easiest to use. Here, we present Rocker, a simple ROC curve visualization tool that can be used for the generation of publication quality images. Rocker also includes an automatic calculation of the AUC for the ROC curve and Boltzmann-enhanced discrimination of ROC (BEDROC). Furthermore, in virtual screening campaigns it is often important to understand the early enrichment of active ligand identification, for this Rocker offers automated calculation routine. To enable further development of Rocker, it is freely available (MIT-GPL license) for use and modifications from our web-site (http://www.jyu.fi/rocker).

**Graphical Abstract:**

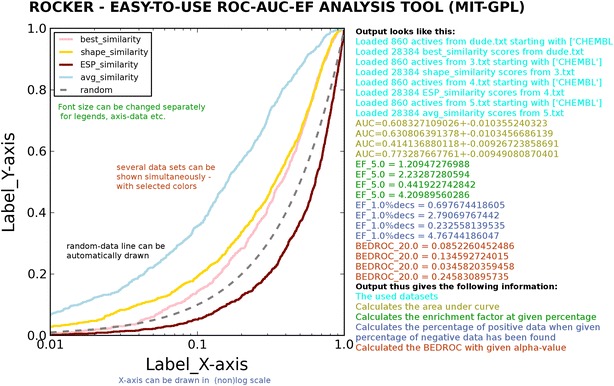

## Background

In early stages of drug discovery, virtual screening (VS) offers an attractive way to identify hit molecules for the target protein. Although there are a wide variety of tools to perform VS, it is necessary to validate their efficiency in separation of active ligands from inactive molecules. One issue that has helped validation significantly is the appearance of databases of ligand binding data, e.g. ChEMBL [1], and molecule collections, where not only active ligands but also decoy molecule sets are available, e.g. DUD [2], DUD-e [3], and DEKOIS [4, 5]. The other important issue in VS efficiency is the numerical and visual illustration of how well the VS method works. For this, two issues are typically calculated: (1) area under curve (AUC) for the receiver operation characteristics (ROC), and (2) early enrichment, e.g. upon the top 1 %. There are many possibilities to avoid the bias in the ROC AUC analysis [6, 7]. The ROC AUC value itself does not directly give detailed information about the early enrichment, but the visualization of it does. Especially, plotting ROC as a semi-logarithmic curve improves the readability a lot. Also weighting each active based on the size of the lead series to which it belongs [6] or incorporating the notion of early recognition into the ROC metric formalism [7] can give useful information about the enrichment of the active molecules. When ROC AUC value is reported with early enrichment, already the two numbers give a good idea for the quality of the used method to separate true positives from false positives.

For the ROC AUC visualization there are many tools [8], e.g. pROC [9], ROCR [10], Pcvsuite [11] that work on top of widely used R-package, and some of them contain sophisticated ROC comparisons for the analysis of medical data. Furthermore, there are web-based tools, such as jrocfit (http://rad.jhmi.edu), and standalone tools like MedCalc [12]. However, as all of these tools have been developed for calculation and comparison of medical data, they do not continue handy tools for VS efficiency analysis. Furthermore, the VS efficiency data is used in the comparison of different VS strategies and tools, and as we noticed in our previous study [13], authors have different opinions about the methods and types of calculations that should be employed with VS analysis. Motivated from this, we introduce a very user-friendly tool called Rocker dedicated for the VS analysis. Rocker calculates the ROC AUC-values, BEDROC-values [6, 7], draws the curves either as semi-logarithmic or non-logarithmic scale, and calculates the enrichment at the given percentage with two commonly used ways.

## Implementation

Rocker is written with Python, and requires in addition to that, the Python-matplotlib library, which is typically available through Linux package management tools, e.g. yum in Red Hat and Fedora distributions. The ROC and AUC are calculated using algorithms described by Fawcett [14]. Fawcett has described the algorithms in a clear way utilizing pseudocode. For the conservative estimate of the standard error for the AUC there are several solutions available, from which the commonly used method developed by Hanley and McNeil [15] was implemented into Rocker. Hanley’s nonparametric approach has the advantage of being simple to calculate, and the corresponding accuracy indexes are obtainable even for small sample sizes [16]. Furthermore, the BEDROC-values with varied alpha can be calculated in order to calculate the ROC with weighted early enrichment [7].

Rocker can calculate the enrichment factors in two commonly used ways, in order to make it easier for the user to compare own results with the published ones: (1) for the top X % of the results, (EFX; Eq. 1), and (2) for the top results until X % of the decoy molecules have been found (EFXdec; Eq. 2).

1 $$EFX = \frac{{\frac{{{\text{Ligs}}_{{{\text{X\% }}}} }}{{{\text{Mols}}_{{{\text{X\% }}}} }}}}{{\frac{{{\text{Ligs}}_{\text{all}} }}{{{\text{Mols}}_{\text{all}} }}}}$$

2 $$EFXdec = \frac{LigsX\% dec}{\text{Ligs}_{\rm all}} \times 100$$

In Eq. (1) Ligs_X%_, Mols_X%_, Ligsall and Molsall are the number of the ligands in the top X % of the screened compounds, the number of the molecules in the top X % of the screened compounds, the total number of the screened ligands, and the total number of the screened molecules, respectively. In Eq. (2) Ligs_X%dec_ is the number of the ligands when X % of the decoy molecules have been found and, again, Ligsall is the total number of the screened ligands.

There are some command line options available in Rocker to control the quality and properties of the output figure and to calculate the enrichment factor. The true and false positives can be separated in two ways: (1) true positives have some difference in their names, which is possible to indicate with regular expression, in contrast to false positives; (2) the names of the true positives can be given as list in a separate file. The figure itself can be manipulated in many ways: (1) resolution and size of the image can be adjusted; (2) labels, font and font size, including the location of legend can be modified; (3) X-axis can be drawn with linear or logarithmic scale; (4) axis thickness and tick size can be adjusted; (5) colors, thickness, and line styles (e.g. solid, dashed) of the plotted curves can be changed; (6) “random-selection curve” can be included or excluded. And finally, the enrichment factors can be calculated in two different ways (see above). The AUC is printed if sufficient data is given. If you do not have graphical output available, you can still calculate the AUC-values and enrichment factors by preventing the drawing of ROC.

## Results and discussion

Rocker can be downloaded from http://www.jyu.fi/rocker for linux (rpm), windows, and mac os. Furthermore, Rocker can also be used via simplified web-interface (available at http://www.jyu.fi/rocker) where user can download the text-file that consists the name-field (1st column) and numerical data that describes the activity/fitness/score (column number for this data can be specified). In current web-interface version the names of true positives (or active compounds) should differ from those of false positives (or decoy molecules). Output figure can be drawn either with linear or logarithmic X-axis, ROC can be drawn either with solid or dashed line with option for color selection. Resolution of the figure can be specified. Furthermore, calculation of BEDROC, EF, and EFdec can be performed with wished values.

**Fig. 1:**
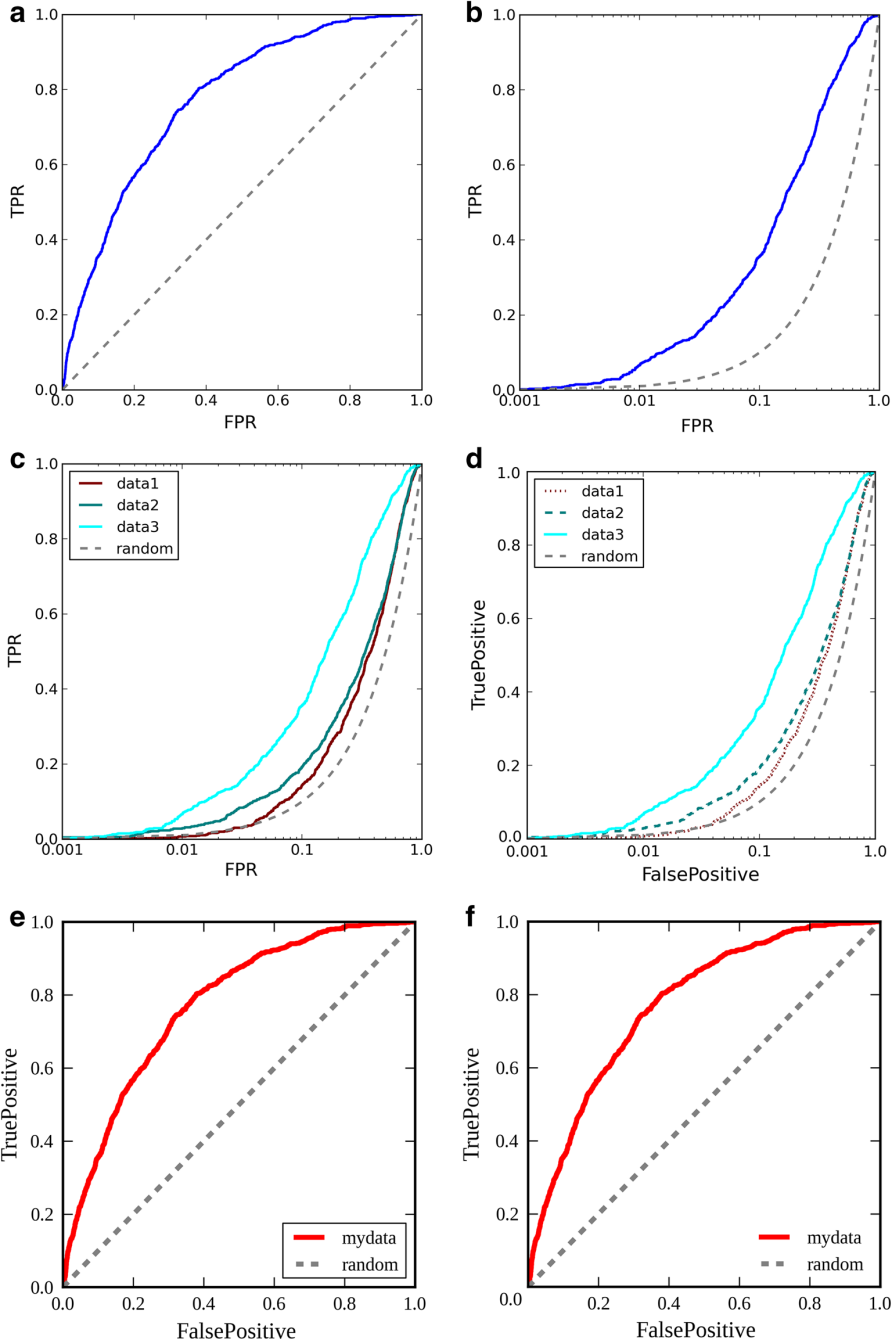
Six example ROCs with different command line options

To visualize the performance of Rocker, here are six example commands, and the figures (Fig. 1) they produce from an example input files (found from Rocker homepage):

(A) rocker dude.txt -an CHEMBL -c 5 -s 5 5 -p Fig1A.png
  This is simple example, where the names of all active ligands begin with CHEMBL (-an; Note that the names of inactives cannot begin with CHEMBL then). The 5th column has the score/fitness-value that is compared (-c; molecule names in 1st column). The produced image has is sized (5*5) inches (-s). Finally, the prepared figure is called Fig. 1a (-p)

(B) rocker dude.txt -an CHEMBL -c 5 -s 5 5 -lp 0.001 -p Fig1B.png
  Similar as in (A) but the X-axis is drawn in logarithmic scale, beginning from 0.001 (−lp).

(C) rocker 2.txt 3.txt 5.txt -an CHEMBL -s 5 5 -li data1 data2 data3 random -l 0 -lp 0.001 -cl maroon teal cyan -p Fig1C.png
  In this example, three curves are drawn from three different files (2.txt, 3.txt, and 5.txt). Legends for each curve are written (–li) and the position of legend-box is indicated (-l). The colors of curves are given (-cl).

(D) rocker 2.txt 3.txt 5.txt -an CHEMBL -s 5 5 -li data1 data2 data3 random -l 0 -lp 0.001 -cl maroon teal cyan -st dotted dashed solid -la FalsePositive TruePositive -las 15 -ts 15 -p Fig1D.png
  Otherwise as (C) but the line styles for curves are changed (-st), axis labels are defined (-la), and their font size is set (-las), label size for tick numbers is defined (-ts)

(E) rocker dude.txt -an CHEMBL -s 5 5 -c 5 -li mydata random -l 4 -les 15 -cl red -lw 4 -la FalsePositive TruePositive -las 15 -ts 15 -aw 2 -f “Liberation Serif” -p Fig1E.png
  Here, the new elements are changed font size for legend (-les), linewidth of the ROC curves (-lw), width of axes (-aw), and defined font (-f).

(F) rocker dude.txt -an CHEMBL -s 5 5 -c 5 -li mydata random -l 4 -les 15 -cl red -lw 4 -la FalsePositive TruePositive -las 15 -ts 15 -aw 2 -f “Liberation Serif” -no -a ‘\-0.08,\-0.05,0.0’ -as 15 -kw “legend:{frameon:False}” -EFd 1 -EF 1 -BR 20 -p Fig1F.png
  Here, the origo is not drawn, i.e. values 0.0 for X- and Y-axis (-no), but one 0.0 is written to position coordinate position -0.08,-0.05 (-a), which font size is set to 15 (-as), the box for legend is not drawn (-kw). Furthermore, the enrichment factors (both types) are calculated at top-1% (-EFd, -EF), bedrock is calculated with alpha-value of 20 (-BR).

As an example, the output from command (F) (as well as output from web-interface), looks like this:

- Loaded 860 actives from dude.txt starting with [‘CHEMBL’]
- Loaded 28384 average scores from dude.txt
- AUC = 0.773287667761 + −0.00949080870401
- Plotting ROC Curve…
- EF_1.0 = 5.22961021946
- EF_1.0%decs = 4.76744186047
- BEDROC_20.0 = 0.245830895735

## Conclusions

As is, Rocker offers a highly useful, easy-to-use tool for ROC analysis in VS, including calculations of AUCs and early enrichments. Although authors sincerely hope that the future developments are made available for the other users as well, that is not required by the license.

## Availability and requirements

- Project name: Rocker.
- Project home page: http://www.jyu.fi/rocker.
- Operating system: Platform independent.
- Programming language: Python.
- Other requirements: Python-matplotlib.
- License: MIT-GPL.
- Any restrictions to use by non-academics: none.

## Abbreviations

AUC: area under curve
BEDROC: Boltzmann-enhanced discrimination of receiver operating characteristics
ROC: receiver operating characteristics
VS: virtual screening
